# Apigenin protects against ischemic stroke by increasing DNA repair

**DOI:** 10.3389/fphar.2024.1362301

**Published:** 2024-04-30

**Authors:** Niu Ping, Kuiyang Zuo, Jiahan Cai, Chunshu Rong, Ziqiao Yu, Xu Zhang, Gaihua Wang, Chunyu Ma, Huirong Yang, Jinhua Li, Xu Wang, Dexi Zhao

**Affiliations:** ^1^ Department of Encephalopathy, Hospital of Changchun University of Chinese Medicine, Changchun, Jilin, China; ^2^ School of Public Health, Jilin University, Changchun, Jilin, China; ^3^ Traditional Chinese Medicine College, Guangdong Pharmaceutical University, Guangzhou, Guangdong, China; ^4^ College of Traditional Chinese Medicine, Changchun University of Chinese Medicine, Changchun, Jilin, China

**Keywords:** Apigenin, ischemic stroke, parthanatos, homologous recombination repair, non-homologous end link repair

## Abstract

**Background and Objective:**

Oxidative stress is an important pathological process in ischemic stroke (IS). Apigenin (APG) is a natural product with favorable antioxidative effects, and some studies have already demonstrated the antioxidative mechanism of APG in the treatment of IS. However, the mechanism of APG on DNA damage and repair after IS is not clear. The aim of this study was to investigate the mechanism of APG on DNA repair after IS.

**Methods:**

Male Sprague-Dawley rats were used to establish a model of permanent middle cerebral artery occlusion (pMCAO) on one side, and were pre-treated with gavage of APG (30, 60, or 120 mg/kg) for 7 days. One day after pMCAO, the brain tissues were collected. Cerebral infarct volume, brain water content, HE staining and antioxidant index were analyzed to evaluated the brain damage. Molecular Docking, molecular dynamics (MD) simulation, immunohistochemistry, and Western blot were used to explore the potential proteins related to DNA damage repair.

**Results:**

APG has a low binding score with DNA repair-related proteins. APG treatment has improved the volume of cerebral infarction and neurological deficits, reduced brain edema, and decreased parthanatos and apoptosis by inhibiting PARP1/AIF pathway. In addition, APG improved the antioxidative capacity through reducing reactive oxygen species and malondialdehyde, and increasing glutathione and superoxide dismutase. Also, APG has reduced DNA damage- and cell death-related proteins such as PARP1, γH2A.X, 53BP1, AIF, cleaved caspase3, Cytochrome c, and increased DNA repair by BRCA1 and RAD51 through homologous recombination repair, and reduced non-homologous end link repair by KU70.

**Conclusion:**

APG can improve nerve damage after IS, and these protective effects were realized by reducing oxidative stress and DNA damage, and improving DNA repair.

## 1 Introduction

Stroke is a common disease with high morbidity and disability worldwide and is also one of the leading causes of death worldwide. Currently, about 80 million people suffer from stroke globally, and the burden of stroke has become the second highest in the world (Feigin et al., 2022). Strokes are divided into hemorrhagic stroke and ischemic stroke (IS), with IS accounting for about 80% of all stroke. However, the availability of drugs for the treatment of IS is very limited. The only approved medication of IS by US Food and Drug Administration is recombinant tissue plasminogen activator, but a strict contraindication (hemorrhagic transformation) and time window has limited its use. In addition, the intense oxidative stress induced by ischemia-reperfusion underlies the pathology leading to severe complications.

Oxidative stress plays an important role in the pathological process of IS. After the onset of IS, large amounts of ROS are released, leading to oxidative DNA damage, which is a serious consequence of oxidative stress (Wang et al., 2006), and can lead to DNA double-strand breaks (DSBs) and finally lead to neuronal cell death through a variety of mechanisms (Martin, 2008). In the face of DNA damage, there exists a well-established cellular response mechanism called the DNA damage response, including homologous recombination (HR) and non-homologous end-joining (NHEJ) to repair these damage (Mao et al., 2008; Shrivastav et al., 2008). Among them, P53-binding protein 1 (53BP1) (Lu et al., 2019) plays a key regulatory role in DSB repair signaling selection, which is involved in the regulation of HR and NHEJ-related proteins ATP-dependent DNA helicase ku70 (KU70) (Kim et al., 2014), Breast cancer type 1 susceptibility protein (BRCA1) (Lou et al., 2003), DNA repair protein RAD51 homolog 1 (RAD51) (Raderschall et al., 2002) and so on. Additionally, poly (ADP-ribose) polymerase 1 (PARP1), which accounts for more than 90% of the PARPs superfamily, is closely related to DNA repair signaling and cell death (Pieper et al., 1999). Increased PARP-1 expression after IS triggers the nuclear translocation of apoptosis inducing factor (AIF), which subsequently induces chromatin lysis and caspase-independent cell death through interaction with histone variant H2AX (Yu et al., 2006; Artus et al., 2010). This caspase-independent cell death mediated by the PARP/AIF pathway has been named parthanatos. Ischemia-induced neuronal DNA damage during oxidative stress is closely associated with parthanatos.

As a DNA damage receptor, about 90% of polyADP ribose (PAR) is produced in response to DNA damage or oxidative stress (Liu S. Q. et al., 2022). DNA damage is a response to cerebral ischemia, including active and passive DNA damage after ischemic brain injury. Active DNA damage is mediated by DNA endonucleases, also known as endonuclease-mediated DNA damage. The most studied active DNA damage is apoptotic DNA fragmentation, which is characterized by DNA double-strand breaks (DSBs) (Chen et al., 1997). DNA fragmentation involves a cascade of cellular self-destruction that is often irreversible. Two endonucleases, caspase-activated deoxyribonucleases and apoptosis inducing factor (AIF), are considered to be the major endonucleases during DNA fragmentation (Li et al., 2011). However, recent findings suggest that at least two pathways upstream of AIF release are involved: one dependent on upstream bcl-2 family proteins such as Bax and caspases, and the other on PARP-1 (Cregan et al., 2004). Translocation of AIF from the mitochondria to the nucleus was identified as a critical step during PARP-1 cell death (Cho and Toledo-Pereyra, 2008). PARP1 inhibition attenuated AIF migration from mitochondria to the nucleus and protected neurons from death after ischemic stroke (Beneke, 2008).

Apigenin (APG) is a natural product widely distributed in nature (green celery heart, Chinese celery), and has the best antioxidative effect among the ten flavonoids (Shrivastav et al., 2008). In a previous study, we have reviewed the therapeutic mechanisms of APG against IS (Wang et al., 2022). The protective effects of APG on IS include anti-inflammation, anti-oxidation and anti-apoptosis. However, whether the antioxidative protection of APG against IS is *via* DNA repair remains unclear. During post-ischemic oxidative stress, we hypothesized that the neuroprotective effects of APG are related to the regulation of DNA damage repair, parthanatos and apoptosis.

In this study, we have constructed a permanent middle cerebral artery occlusion (pMCAO) model after pretreating rats with APG for 7 days. DNA damage repair, apoptosis, and parthanatos were detected in ischemic brain tissues. We found that the protein expression of DNA repair, parthanatos and apoptosis was increased, and DNA repair-related proteins were decreased in the ischemic semi-dark band brain tissue of pMCAO. This phenomenon was reversed by different doses of APG, especially in the medium dose group (60 mg/kg). In addition, we found that APG was involved in regulating DNA repair to rescue neuronal survival in the ischemic semi-dark band. Our study reveals a novel mechanism of APG in the treatment of IS through antioxidant therapy and increasing DNA repair, which provides a theoretical basis for the clinical translation of APG.

## 2 Materials and methods

### 2.1 Reagents

APG and 2,3,5-Triphenyltetrazolium chloride (TTC) were purchased from Meryer Chemical Technology Co. Ltd (Shanghai), and malondialdehyde (MDA) assay kit, total superoxide dismutase (SOD) assay kit, and glutathione (GSH) assay kit were purchased from Nanjing Jiancheng Bioengineering Institute. Nylon wire with a rounded tip (diameter 0.36 mm) was purchased from BEIJING CINONTECH CO.

### 2.2 Experiment animals

Adult male Sprague-Dawley (SD) rats (250 ± 30 g) were purchased from Liaoning Changsheng biotechnology Co. Ltd. The rats were housed in the Animal Experiment Center, School of Public Health, Jilin University, China. All operations of rats were complied with the National Institutes of Health Guide for the Care and National Research Council of the National Academies. Rats were kept under stable conditions with temperature around 23°C ± 1°C, 12 h light/12 h dark cycle, free access to food and water, and air humidity at 60%–70%. This experiment was approved and supervised by the Animal Experimentation Committee of Changchun University of Traditional Chinese Medicine (ID: 2023158).

### 2.3 Establishment of pMCAO model and animal grouping

The rats were randomly divided into five groups, including the sham operation group (Control group), the model group (pMCAO group), the APG low-dose group (30 mg/kg, L group), the APG medium-dose group (60 mg/kg, M group), and the APG high-dose group (120 mg/kg, H group). The dosage of APG was determined based on previous studies (Wang et al., 2022). The treatment group was pretreated with APG through gavage for 7 days consecutively, and the control and model groups were given equal amounts of sterile saline. All animals were given free access to food and water in an appropriate environment. Rats were weighed and anesthetized by intraperitoneal injection of sodium pentobarbital (2%, 2 mL/kg), and then fixed in the ventral recumbent position, with a warming pad to maintain the core temperature at approximately 37.0°C throughout the operation. The Longa’s method was used to establish pMCAO model. The skin was incised along the midline of the neck to expose the common carotid artery (CCA), external carotid artery (ECA) and internal carotid artery (ICA). The CCA and ECA were ligated, and the ICA was gently inserted using a round-tipped nylon wire until slight resistance was felt. The rats were executed 24 h later. Rats in the control group underwent the same surgical exposure steps as the model group except for the insertion of nylon wires.

### 2.4 Neurological deficit score

The Longa method was used to score the neurological function of rats in each group at 24 h after the pMCAO model. The scoring criteria were as follows: 0 point, normal neurological function; 1 point, rats were unable to fully extend the contralateral forepaw; 2 points, rats walked in a circle toward the hemiplegic side; 3 points, rats were prone toward the hemiplegic side at rest; 4 points, rats lost consciousness and were unable to walk on their own (Longa et al., 1989).

### 2.5 Analysis of cerebral infarct volume, brain water content and antioxidant index

After 24 h of modeling, the rats were anesthetized and executed by severing the head, and the brain tissues were taken and frozen at - 20°C for 15 min, and then the brain tissues were cut into 2 mm-thick coronal sections and stained with 2% TTC solution at 37°C for 20 min, and images were taken and analyzed by ImageJ software.

Ischemic hemisphere of cerebral edema was determined using the wet/dry method. The formula for the calculation is [(wet weight - dry weight)/wet weight] × 100%.

Brain tissues from the ischemic semi-dark zone were collected as test samples. ROS were detected by probes, and MDA, GSH and SOD were detected by kits according to the manufacturer’s procedure. Briefly, 10% tissue homogenate was first prepared, protein concentration was determined and experiment followed with the instructions.

### 2.6 Pathological and immunohistochemical detection of brain tissue

HE staining was performed for histologic examination. Briefly, brain tissues of each group were collected and fixed in 10% paraformaldehyde. Tissue sections were dehydrated, processed in an automated tissue processor and immersed in paraffin. 4 μm paraffin sections were made. HE staining was performed after dewaxing and hydration. Sections were scanned with a scanner.

Immunohistochemical staining was performed on paraformaldehyde-fixed, paraffin-embedded brain tissue sections. Brain sections of 3–4 μ m thickness were incubated with anti-γH2A.X antibody. The primary antibody was incubated at 37 °C for 1 h. Subsequently, the brain slices were incubated with horseradish peroxidase-conjugated secondary antibody at 37 °C for 30 min, and the color reaction was carried out using diaminobenzidine as the chromogen. The final sections were restrained with hematoxylin, dehydrated with graded ethanol and xylene, and images of ischemic semi-dark bands were taken under a light microscope. The integral optical density values of positive cells were analyzed using ImageJ software.

### 2.7 Verification of molecular docking and molecular dynamics (MD) simulation

The 3D conformations of APG were downloaded from PubChem (https://pubchem.ncbi.nlm.nih.gov/), energy minimized using Chem3D software, and saved in pdb format. AIF (PDBID:1M6I), MIF (PDBID:1CA7), H2AX (PDBID:6K1I), PARP1 (PDBID:4L6S), BRCA1 (PDBID:4IFI), RAD51 (PDBID. 7EJC), KU70 (PDBID:1JEY), KU80 (PDBID:6ERH), CASP3 (PDBID:1NMS) and other protein conformations were obtained from RCSB PDB database. The receptor and ligands were pre-processed using Pymol and Autodock tool, and docked using AutodockVina 1.2.3 to screen the best binding conformations based on binding energy.

As the conformations of ligand and receptor are constantly changing during the binding process, the conformational situation of semi-flexible docking is still somewhat different from the actual situation. Therefore, we further used molecular dynamics simulations to explore the stability of the binding between APG and nine proteins. All-atom molecular dynamics simulations were performed using the classical molecular dynamics simulation program GROMACS 2020.3, simulations of protein-ligand complexes were performed based on the Amber 99SB-ILDN force field, and ligand molecular topology files were generated using the programs Antechamber and Acpype. Saline solvation boxes of cubic (cubic) were selected, the closest distance between the system boundary and the complex was set to 1.2 nm, and Na^+^ or Cl^-^ was randomly added to neutralize the system charge. The energy of each system was minimized using a most rapid descent algorithm with a maximum of 50,000 steps. Subsequently, the system was warmed up to 310.15 K with a 100 ps simulation under the normal-variance tether (NVT) and a continuous 100 ps simulation under the isothermal-isobaric tether (NPT). After the system reaches equilibrium, the system bond lengths and bond-forming interactions are constrained with the LINCS algorithm, free dynamics simulations are performed for 50 ns, and finally the trajectories are analyzed using the GROMACS tool and VMD.

### 2.8 Western blot analysis

**FIGURE 1:**
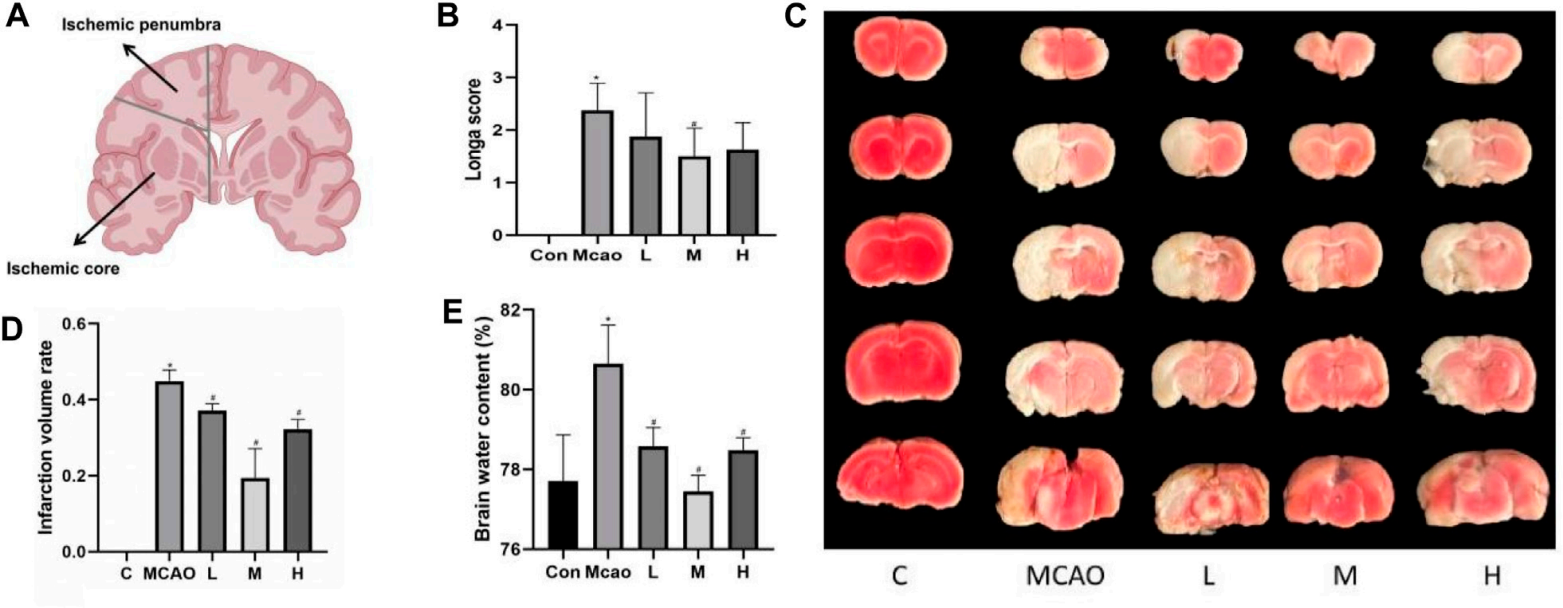
Effect of APG treatment on cerebral infarct, neurological function, and brain edema. **(A)** Diagram of ischemic penumbra and ischemic core. **(B)** Statistical graph of neurological deficit score after 1 day pMCAO. Data are expressed as mean ± SD (n = 10). **(C)** Representative TTC staining of rat brain. **(D)** Infarct volumes (%). Data are expressed as mean ± SD (n = 3). **(E)** Statistical graph of the brain water content. Data are expressed as mean ± SD (n = 4). *p < 0.05, compared with Con; #*p* < 0.05, compared with pMCAO.

The brain tissues of the ischemic penumbra (Figure 1A). Brain tissue was crushed under liquid nitrogen, RIPA lysate was added, and protease inhibitors were added. After lysis on ice for 30min, the samples were centrifuged at 12000 rpm for 10 min, the supernatant was removed, and the precipitate was discarded. The supernatant was removed to measure the protein concentration using the BCA method. Loading buffer was added to unify the protein concentration at 95°–100° and boiled for 10 min 30 μg of protein samples were separated by sodium dodecyl sulfate–polyacrylamide gel electrophoresis (SDS-PAGE) and transferred to polyvinylidene difluoride (PVDF) membrane. Subsequently, the membranes were closed with 5% BSA for 60 min at room temperature and then incubated overnight at 4°C with anti-γH2A.X (1:1000, CST: #7631), anti-PARP1 (1:1000, CST: #94885 and abcam: ab227244), anti-AIF (1:1000, CST: #5318 and Proteintech: 67791-1-Ig), anti-GAPDH (1:10,000, CST: #2118), anti 53BP1 (1:1000, ZEN-BIOSCIENCE: 381816), anti Caspase3 (1:1000, CST: #9661), anti-Cytochrome c (1:1000, CST: #4272), anti-KU70 (1:1000, CST: #4588), anti-BRCA1 (1:1000, abcam: ab238983), and anti-RAD51 (1:1000, CST: #8875). Subsequently, the membranes were washed with TBST and then incubated with horseradish peroxidase-conjugated secondary antibody for 60 min. Blots were visualized using ECL reagent (GE Healthcare, Piscataway, NJ, United States). ImageJ software was applied to analyze the band densities.

### 2.9 Statistical analysis

All experimental data passed Shapiro-Wilk normality test by SPSS 22.0. Data were analyzed using GraphPad Prism. All data were expressed as mean ± standard deviation (SD) and analyzed by one-way analysis of variance (ANOVA) followed by Tukey’s test. Differences were considered statistically significant at *p* < 0.05.

## 3 Results

### 3.1 APG improves neurological deficits, reduces cerebral infarct volume and cerebral water content after pMCAO

As shown in Figure 1A, the rats in the pMCAO group showed significant neurological deficits compared with the control group (*p* < 0.05), and the pretreatment with medium-dose APG was effective in improving the neurological deficits compared with the pMCAO group (*p* < 0.05). Compared with the control group, the cerebral infarct volume (Figures 1B, C) and cerebral edema (Figure 1D) was significantly increased in the pMCAO group (*p* < 0.05), and the infarct volume was significantly reduced in APG treatment groups (*p* < 0.05), with the medium-dose group being the most significant. Compared with the control group, the water content of the left cerebral hemisphere of the rats in the pMCAO group was significantly increased, whereas brain edema was reduced in APG treatment groups compared with the pMCAO group, with the medium-dose APG being the most significant (Figure 1E).

### 3.2 APG has high binding energy and stable binding to the proteins-related to DNA damage repair and parthanatos

**FIGURE 2:**
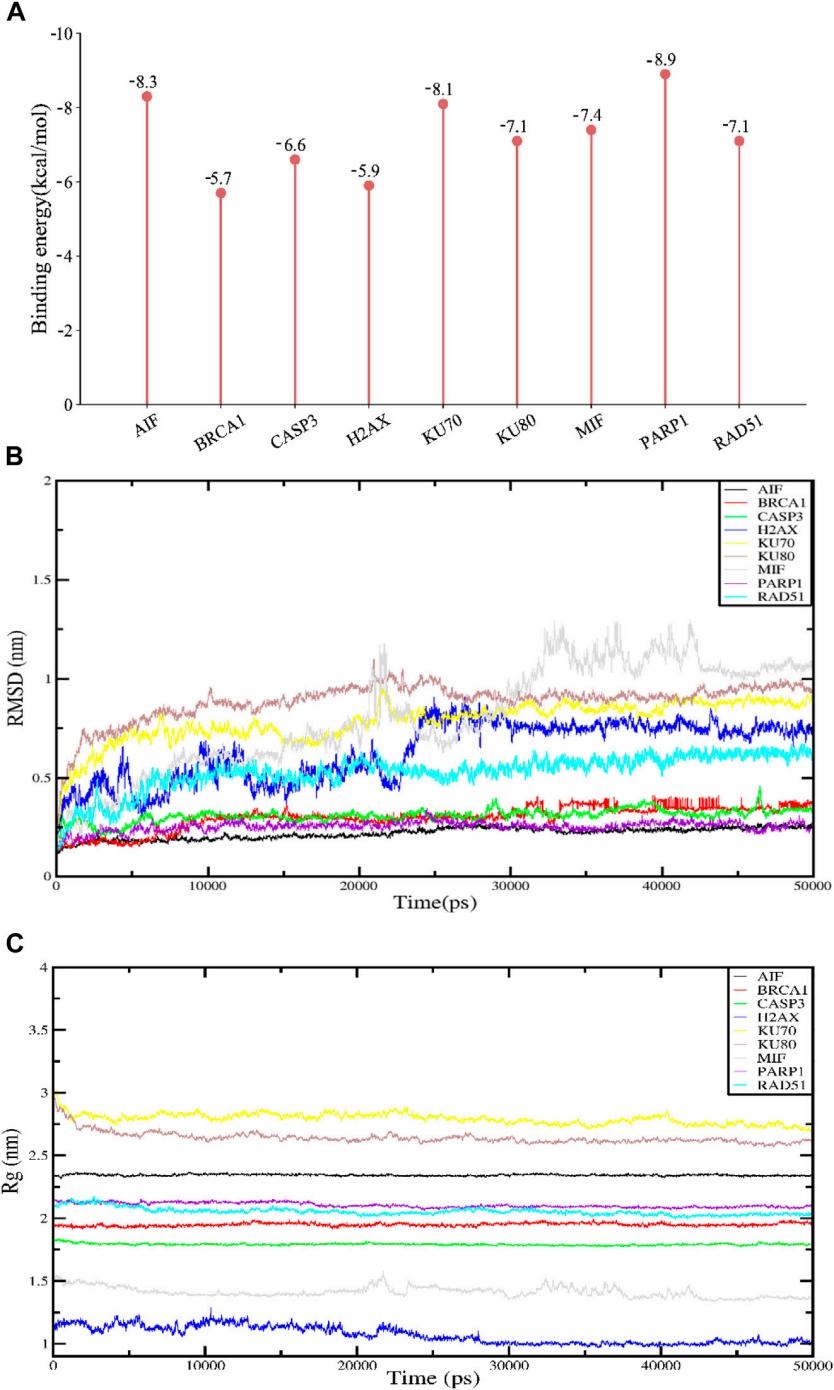
Molecular docking and molecular dynamics simulation results. APG has high binding fractions and stability with all docked proteins. **(A)** Molecular docking binding scores of APG to proteins associated with DNA damage, repair and programmed cell death. **(B)** RMSD results for APG molecular dynamics. **(C)** Rg results for APG molecular dynamics.

By calculating the binding fraction, the molecular docking result of APG with proteins-related to DNA damage repair and parthanatos was predicted to be less than −5.0 kcal/mol, indicating that APG has a strong binding with these proteins. In other words, the lower the binding fraction of the ligand to the receptor, the more stable the binding conformation. As can be seen from Figure 2A, the binding affinity of all the docking results was below - 5 kcal/mol. The free binding scores of the docking results ranged from −5.7 ∼ −8.9 kcal/mol, indicating that APG binds stably to these targets. The lowest binding scores were observed for PARP1 with APG. The Root Mean Square Deviation (RMSD) curve can reflect the degree of fluctuation of the system conformation. As shown in Figure 2B, the RMSD values of the complexed systems of APG and each protein showed some fluctuation and increase in the early stage, which indicated that the conformation changed to some extent relative to the docking initial conformation. However, the RMSD values of the complexes stabilized after a certain period, which indicated that the conformations of the APG-protein complexes did not change significantly, and the APG-protein complexes were able to maintain stable binding. Radius of Gyration (Rg) can characterize the compactness of the system structure. As shown in Figure 2C, the complexes of APG and nine proteins have relatively stable radius of gyration, suggesting that the complexes are conformationally stable and compact, and is in agreement with the results of RMSD. Thus, the molecular dynamics results demonstrated that the relevant proteins have a stable binding conformation with APG.

### 3.3 APG improves histopathology, reduces DNA damage and promotes neuronal survival

**FIGURE 3:**
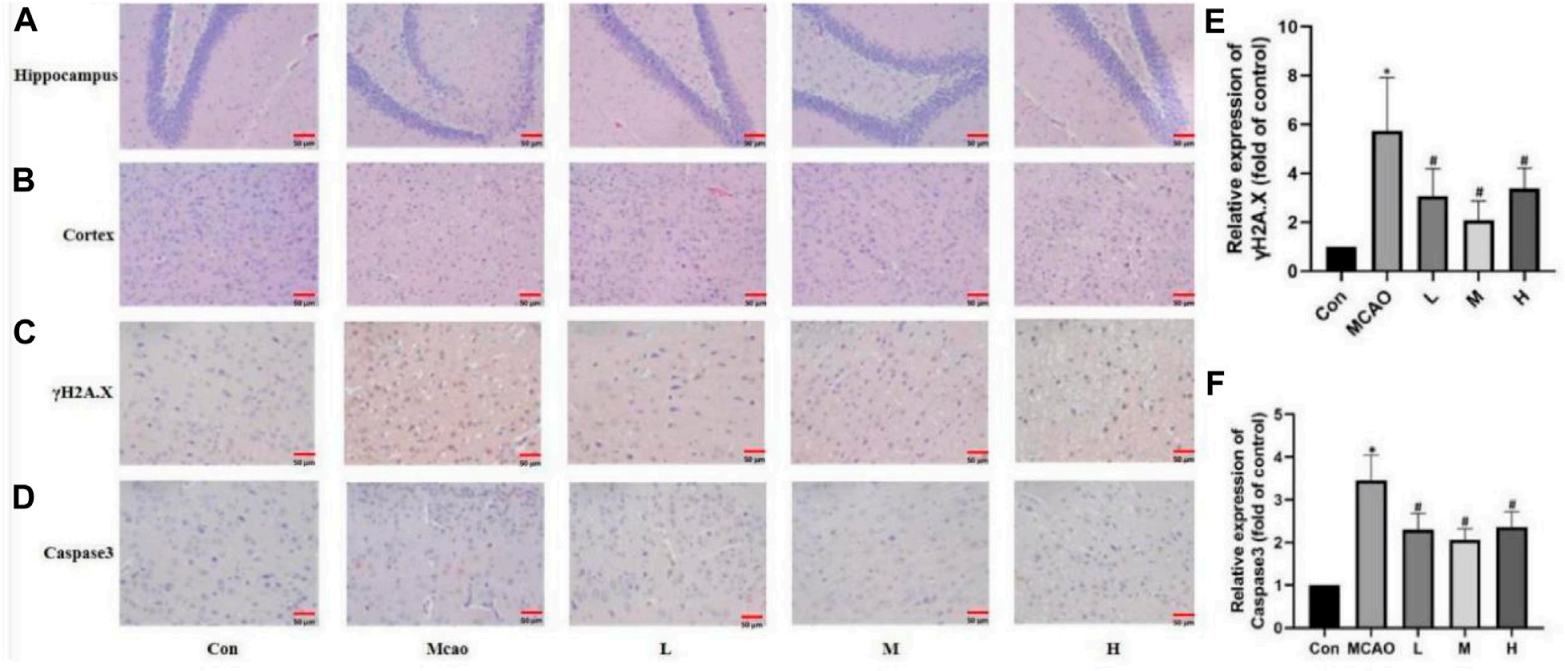
APG on pathological changes after pMCAO. **(A,B)** Representative pathological images of the ischemic penumbra (Hippocampus and cortex). Bar = 50 µm. **(C–F)** Immunostaining photomicrographs of γH2A.X, Caspase3 and quantitative analysis of the integrated optical density. Bar = 50 µm. Data are expressed as mean ± SD (n = 5). *p < 0.05, compared with Con; #*p* < 0.05, compared with pMCAO.

HE staining showed edema, structural vacuolization, nuclear consolidation and increased eosinophils in the ischemic semidarktic band of rats in the pMCAO group, which was effectively improved by APG in all dose groups (Figures 3A, B). Immunohistochemical analysis of γH2A.X staining was performed to observe the DNA damage of neurons in the ischemic semi-dark band. Immunohistochemical results showed that DNA damage was reduced in the APG group compared with the control group, especially in the medium-dose group (Figure 3C, *p* < 0.05).

### 3.4 Anti-oxidative stress and inhibition of DNA damage by APG

**FIGURE 4:**
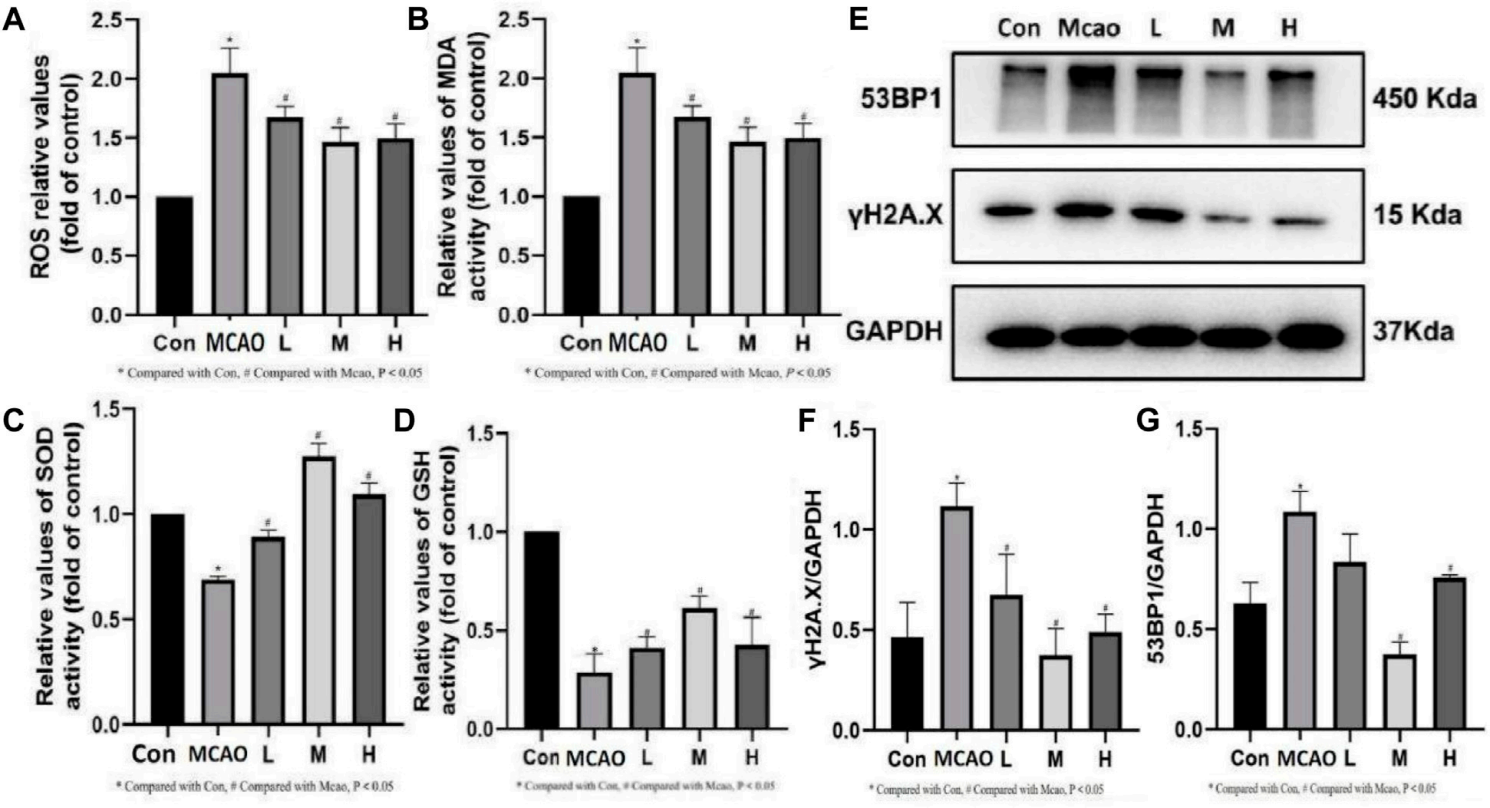
Effect of APG on oxidative stress index and DNA damage. **(A–D)** Statistical graph of ROS, MDA, SOD, and GSH. **(E)** Representative images of Western blot, including 53BP1 and γH2A.X. Data are expressed as mean ± SD (n = 3). **(F,G)** The relative band densities of the target proteins were normalized with GAPDH and normalized to control group, and data are expressed as mean ± SD (n = 3). *p < 0.05, compared with Con; #*p* < 0.05, compared with pMCAO.

The ROS and MDA contents were significantly upregulated and SOD and GSH activities were significantly downregulated in the ischemic penumbra tissue of pMCAO group compared with the control group (*p* < 0.05). The above changes were reversed in the APG treatment groups (*p* < 0.05) (Figures 4A–D). Meanwhile, Western blot results showed that γH2A.X, which is the most important DNA damage marker, was decreased in the APG treatment group especially in the medium dose group (*p* < 0.05) (Figures 4E, F). In addition, APG treatment has effectively downregulated 53BP1 (a key protein regulating NHEJ) protein expression compared with the pMCAO group (*p* < 0.05, Figure 4G).

### 3.5 APG inhibits PARP1-mediated parthanatos and apoptosis

**FIGURE 5:**
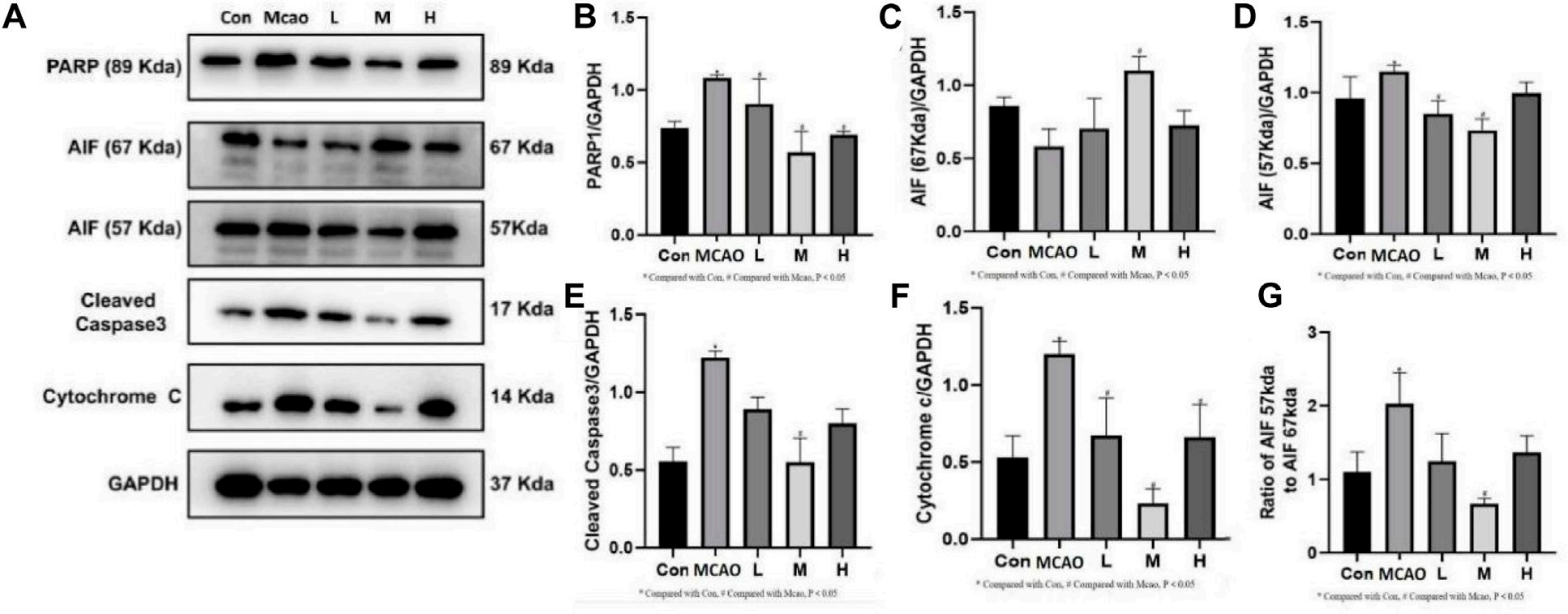
Expression of programmed cell death-related proteins determined by Western blot. **(A)** Representative images of Western blot, including PARP1, AIF, Caspase3 and Cyt c. **(B–F)** The relative band densities of the target proteins were normalized with GAPDH, and data are expressed as mean ± SD (n = 3). **(G)**. Ratio of AIF 57 kda to AIF 67 kda. *p < 0.05, compared with Con; #*p* < 0.05, compared with pMCAO.

The expression of parthanatos- and apoptosis-related proteins was shown in Figure 5A. PARP1 hyperactivation induced by severe DNA damage triggered the initiation of parthanatos in the pMCAO group. pMCAO group showed significantly higher PARP1 expression than the control group (*p* < 0.05) (Figure 5B), and this phenomenon could be effectively reversed by different concentrations of APG treatment (*p* < 0.05). Meanwhile, AIF (67Kda) was significantly decreased in the pMCAO group (*p* < 0.05) than the control group (Figure 5C), and this phenomenon was reversed by medium and high doses of APG. On the contrary, the total content of AIF (57Kda) was significantly higher in the pMCAO group than in the control group (*p* < 0.05, Figure 5D), while the expression of AIF (57Kda) was significantly downregulated in the APG treatment groups (*p* < 0.05). Ratio of AIF 57 kda to AIF 67 kda as shown in Figure 5G. This suggests that APG can reduce apoptosis by inhibiting the entry of AIF into the nucleus. Apoptosis, as the most common mode of cell death, was also examined (Figures 5E, F). Cleaved caspase3 and Cytochrome c expression was significantly increased in pMCAO group in comparison with control group. Both cleaved caspase3 and Cyt c expression were downregulated in the ischemic penumbra brain tissue of the pMCAO model after treatment with different concentrations of APG. This phenomenon was particularly evident in the medium-dose APG group.

### 3.6 APG promotes homologous recombination repair but not non-homologous end linkage

**FIGURE 6:**
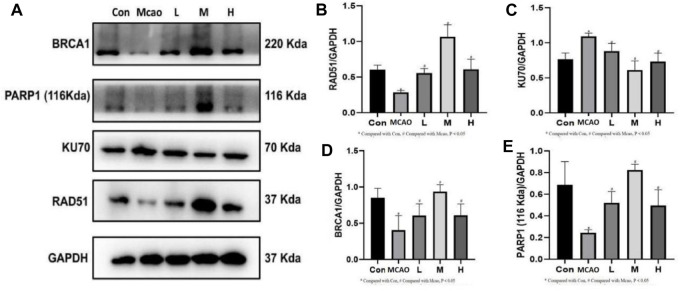
DNA repair-related protein expression determined by Western blot. **(A)** Representative images of WB, including BRCA1, PARP1, KU70 and RAD51. **(B–E)** The relative band densities of the target proteins were normalized with GAPDH, and data are expressed as mean ± SD (n = 3). *p < 0.05, compared with Con; #*p* < 0.05, compared with pMCAO.

The expression of DNA repair-related proteins was shown in Figure 6A. DNA damage-induced activation of PARP1 triggers DNA repair and promotes the survival of damaged cells. pMCAO group showed significantly lower PARP1 expression than the control group (*p* < 0.05, Figure 6B), suggesting that the DNA repair was reduced, which could be effectively reversed by treatment with different concentrations of APG (*p* < 0.05). The repair of NHEJ is prone to errors, causing gene mutations and even cell death. kU70 protein expression was increased in the pMCAO group compared with the control group (*p* < 0.05, Figure 6C). KU70 expression decreased after APG treatment at different concentrations, especially in the medium-dose group (*p* < 0.05). On the contrary, the expression of HR repair-related proteins BRCA1 and RAD51 was increased after APG treatment (*p* < 0.05, Figures 6D,E).

## 4 Discussion

IS is one of the leading causes of death worldwide and the second largest global burden of disease (Feigin et al., 2022). Oxidative stress is one of the important pathological mechanisms of IS. APG possesses the best ROS scavenging function among 10 flavonoids (Guo et al., 2014). However, the molecular mechanism of APG in treating IS through antioxidant therapy remains unclear. In this study, we firstly performed molecular docking and MD simulations of APG with DNA repair- and parthanatos-related proteins. The molecular docking and MD results showed that these proteins have low binding scores and stable binding conformations with APG. These proteins were subsequently validated in our *in vivo* experiments. Our study revealed elevated ROS level, MDA content, and protein expression of PARP1, γH2A.X, 53BP1, AIF, cleaved caspase3, Cytochrome c, and KU70, as well as decreased GSH content, SOD activity and protein expression of BRCA1 and RAD51 in pMCAO group. This phenomenon was reversed by pretreatment with different concentrations of APG and was most pronounced in the medium concentration of APG (60 mg/kg). APG treatment improved neurological deficits, cerebral infarct area, and cerebral water content in rats after pMCAO.

The brain is more susceptible to oxidative damage than other tissues because it has a higher oxygen-consuming and lipid content (Wang et al., 2023). In addition, there is virtually no low-energy reserves in brain, and its energy expenditure is entirely dependent on a continuous vascular supply of oxygen and glucose (Salim, 2017; Bailey, 2019). Increased ROS and MDA and decreased SOD and GSH in ischemic penumbra brain tissue after pMCAO lead to a disruption of antioxidant homeostasis (Elsayed et al., 2020). DNA damage is a serious consequence of oxidative stress and can cause neuronal cell death (Martin, 2008). The most severe case of DNA damage is DSB (Argunhan et al., 2021), where the serine 139 of histone H2AX is rapidly phosphorylated to produce phosphorylated H2AX, which is γH2A.X (Zeng et al., 2021). Both Western blot and immunohistochemistry results confirmed the increased γH2A.X expression after pMCAO. While, the expression of γH2A.X and PARP1 decreased after APG treatment, suggesting that APG attenuated DNA damage, which might in turn reduce parthanatos. APG reduces DNA damage in the ischemic penumbra of the brain through antioxidant.

PARP1 is the most abundant and representative enzyme of the PARP family and is important in repairing DNA damage and maintaining energy homeostasis (Liu S. Q. et al., 2022; Pinton et al., 2023). The first function of PARP1 is to mediate parthanatos (Huang et al., 2022). In many cells, the AIF 67 kDa precursor protein is the predominant form of AIF present. After entering the mitochondrial membrane space it becomes the mature 57 kDa AIF. Parthanatos is mediated by PARP1 for AIF entry into the nucleus and synergizes with macrophage migration inhibitory factor (MIF) to cleave DNA (Wang et al., 2016), i.e., the PARP1/AIF pathway (Liu L. B. et al., 2022). APG reversed the increased expression of PARP1 and AIF after pMCAO. Thus, APG protects against IS in part by inhibiting the PARP1/AIF pathway and improvement of the 67 kda and 57 kda ratios of the AIF. Similarly, APG also decreased the expression of apoptosis-related proteins cleaved caspase 3 and Cyt c, which was higher in the pMCAO group. Therefore, APG protects IS mainly by inhibiting parthanatos and apoptosis.

The second function of PARP1 is to mediate DSB repair (Yang et al., 2023), including NHEJ and HR. Expression of DNA repair proteins after DNA damage can determine the cellular outcomes (Singh et al., 2014). The NHEJ repair pathway is fast and non-precise, while the HR repair pathway is complex and precise (Radhakrishnan et al., 2014). We found that DNA repair protein PARP1 was decreased and 53BP1 expression was increased after pMCAO. 53BP1 promoted DSB repair through NHEJ (Lee et al., 2009). In contrast, 53BP1 expression was decreased in ischemic penumbra brain tissue after APG treatment. This suggests that APG may be involved in DNA repair through HR. Western blot detection of HR repair-related proteins BRCA1 and RAD51 showed both proteins significantly increased after APG treatment. On the contrary, the expression of NHEJ repair-related protein KU70 was decreased. KU70 is the representative protein of NHEJ (Zhu et al., 2014). These results suggest that APG involvement in DSB repair may be more dependent on HR than NHEJ. This needs to be further verified in future rescue experiment.

**FIGURE 7:**
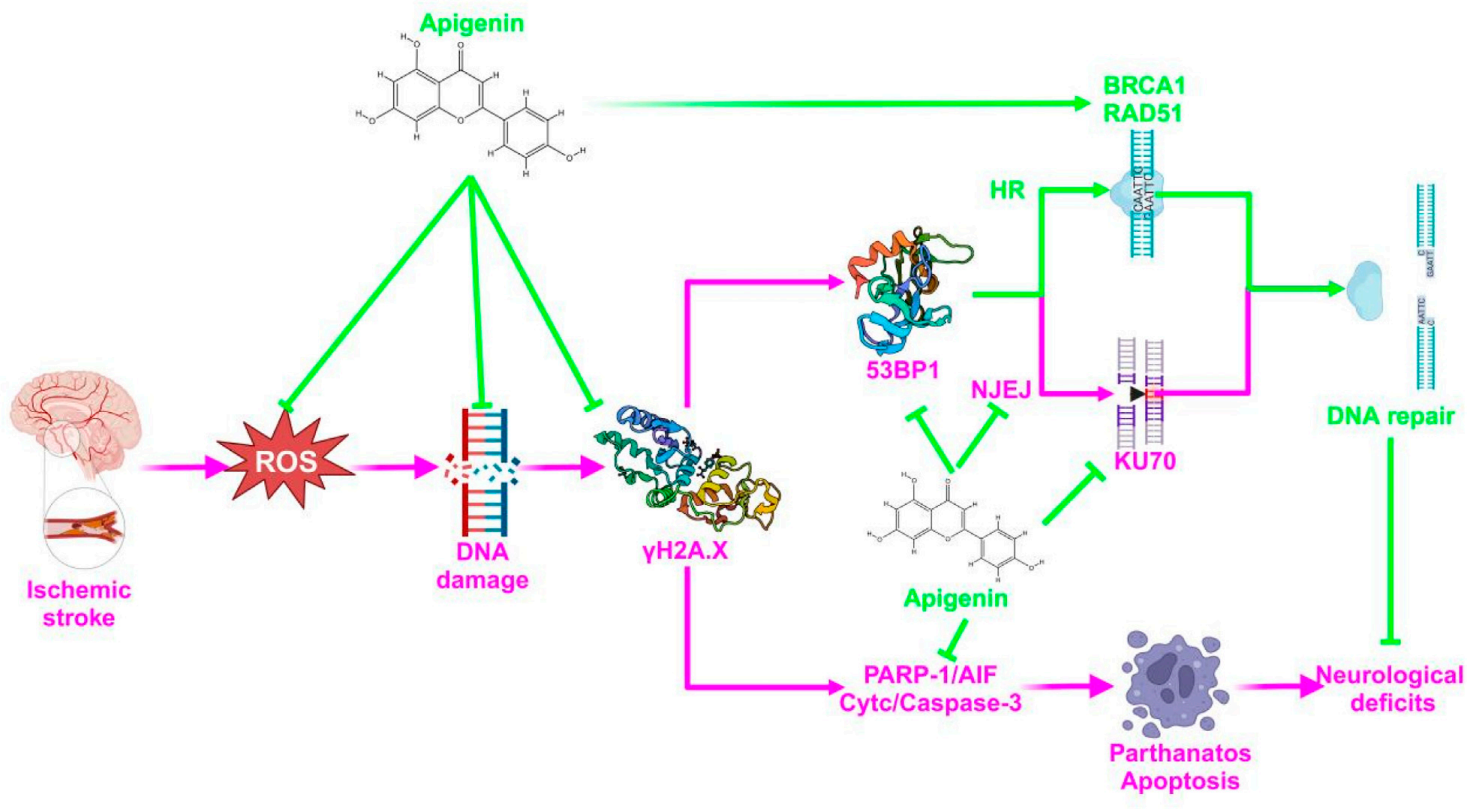
A diagrammatic sketch showing the protective effect of APG on pMCAO. The green color represents the therapeutic effect and the pink color represents the mechanism of the pathology.

The above results suggest that APG bi-directionally regulates PARP1 to reduce nerve damage caused by pMCAO. On one hand, APG reduced PARP1 (89Kda) to inhibit PARP1/AIF pathway, DNA damage, and apoptosis to reduce neural cell death. On the other hand, APG increased PARP1 (116Kda) to improve DNA repair, and this repair may be more in favor of HR repair rather than NHEJ repair. The specific mechanism of APG against IS was proposed and shown in Figure 7. Inevitably, our study has some drawbacks, such as a short pMCAO time and lacks of rescue experiment. In addition, 120 mg/kg of APG may carry a risk of false-positive results.

## 5 Conclusion

In summary, the present study confirmed the novel mechanism of APG in the treatment of IS through antioxidative effects. Moreover, this protective effect of IS is achieved through DNA repair. The future needs more experiments to find the APG through DNA repair mechanisms. This study provides new insights into the mechanism of APG in the treatment of IS. Therefore, APG has the potential to be used as a therapeutic agent for IS. Our study provides a theoretical basis for the clinical translation of APG.

## Data Availability

The original contributions presented in the study are included in the article/supplementary material, further inquiries can be directed to the corresponding authors.
