# Corrigendum: Apigenin protects against ischemic stroke by increasing DNA repair

**DOI:** 10.3389/fphar.2025.1562125

**Published:** 2025-02-17

**Authors:** Niu Ping, Kuiyang Zuo, Jiahan Cai, Chunshu Rong, Ziqiao Yu, Xu Zhang, Gaihua Wang, Chunyu Ma, Huirong Yang, Jinhua Li, Xu Wang, Dexi Zhao

**Affiliations:** ^1^ Department of Encephalopathy, Hospital of Changchun University of Chinese Medicine, Changchun, Jilin, China; ^2^ School of Public Health, Jilin University, Changchun, Jilin, China; ^3^ Traditional Chinese Medicine College, Guangdong Pharmaceutical University, Guangzhou, Guangdong, China; ^4^ College of Traditional Chinese Medicine, Changchun University of Chinese Medicine, Changchun, Jilin, China

**Keywords:** Apigenin, ischemic stroke, parthanatos, homologous recombination repair, non-homologous end link repair

In the published article, there was an error in the author list. The footnote “^**†**^These authors have contributed equally to this work” was erroneously included in relation to the authors **Ping Niu** and **Kuiyang Zuo**. The corrected author list appears above.

The authors apologize for this error and state that this does not change the scientific conclusions of the article in any way. The original article has been updated.

